# A High-Throughput Screening Method for Determining the Optimized Synthesis Conditions of Quinoxaline Derivatives Using Microdroplet Reaction

**DOI:** 10.3389/fchem.2020.00789

**Published:** 2020-09-10

**Authors:** Yanmei Yang, Junmin Liu, Zhenzhen Chen, Weihua Niu, Ran Li, Le Niu, Peng Yang, Xiaoyan Mu, Bo Tang

**Affiliations:** Key Laboratory of Molecular and Nano Probes, Collaborative Innovation Center of Functionalized Probes for Chemical Imaging in Universities of Shandong, College of Chemistry, Chemical Engineering and Materials Science, Shandong Normal University, Ministry of Education, Jinan, China

**Keywords:** microdroplets, screening conditions, quinoxaline derivatives, mass spectrometry, scale-up reaction

## Abstract

Quinoxaline derivatives demonstrate many distinguished chemical, biological, and physical properties and have a wide application in dyes, electroluminescent material, organic semiconductors, biological agents, etc. However, the synthesis of quinoxaline still suffers from several drawbacks, for instance, longer reaction time, unsatisfactory yields, and use of metal catalysts. Here, utilizing microdroplet-assisted reaction, we demonstrate the successive synthesis of several important quinoxaline derivatives. For case studies of 1H-indeno [1, 2-b] quinoxaline and 3,5-dimethyl-2-phenylquinoxaline, the present microdroplet approach can complete in milliseconds and the conversion rate reached 90% without adding any catalyst, which is considerably quicker and higher than conversional bulk-phase reactions. When combined with MS detection, high-throughput screening of the optimal reaction conditions can be achieved. Several impacts of droplet volume, reaction flow rate, distance from the MS inlet, spray voltage, and flow rate of the auxiliary gas can be screened on-site quickly for enhanced reaction speed and yields. More importantly, this platform is capable to be used for the scaled-up microdroplet synthesis of quinoxaline diversities. Considering the facile, economic, and environmentally friendly features of the microdroplet approach, we sincerely hope that the current strategy can effectively promote the academic research and industrial fabrications of functional quinoxaline substances for chemical, biological, and pharmaceutical application developments.

## Introduction

As one of the most important nitrogen heterocycles, quinoxaline moiety is widely recognized to be the main nuclei for the synthesis of numerous biologically active compounds (Ajani, 2014). Numerous quinoxaline derivatives, such as levomycin, actinoleutin, and echinomycin, show high biological activities and are used as therapeutic agents (Bailly et al., 1999; Seitz et al., 2002). Further, these compounds are important intermediates for the industrial fabrication of functional substances such as organic dyes, electroluminescent materials, and organic semiconductors (Dailey et al., 2001; Sonawane and Rangnekar, 2002; Justin Thomas et al., 2005). Due to these distinguished chemical, biological, and physical features of quinoxalines, there has been tremendous interest in devising a simple and efficient method for the synthesis of such structures (Kamal et al., 2006). For instance, Wang et al. reported the synthesis of quinoxaline acids from 2,3-diaminobenzoic acid and aryl glyoxals in ethanol (Wang et al., 2002). This approach has achieved a high yield of 93% and has been successfully used for synthesizing the inhibitor of Topoisomerase I/II. Meshram et al. reported an environmentally friendly and catalyst-free method for the one-pot synthesis of quinoxaline derivatives using ionic liquid at the reaction solvent (Meshram et al., 2010). In addition to this, numerous efforts have been paid on the synthesis of quinoxaline moieties.

Among various works, one of the most commonly used methods is the condensation reaction between 1,2-diamine and 1,2-dicarbonyl compounds. For instance, Lassagne et al. demonstrated the synthesis of quinoxaline derivatives from 1,2-arylenediamines and 1,2-dicarbonyl compounds using ammonium bifluoride as catalyst (Lassagne et al., 2015). In addition, Bandyopadhyay et al. reported that usage of a microwave can significantly improve the condensation of 1,2-diamines with 1,2-dicarbonyl compounds via iodine catalysis and reached high yield (Bandyopadhyay et al., 2010).

Although the aforementioned works have reached good yields, these methods still suffered from several drawbacks, including high reaction temperature, usage of toxic/cost solvent or catalysts, and long reaction time (Subran and Paira, 2017). The need to discover the most facile, economic, and environmentally acceptable method has placed extreme pressure on the development chemist. This has become particularly acute in the pharmaceutical area where combinatorial chemistry, parallel synthesis, and high-throughput screening have accelerated the promotion of compounds into clinical development. In view of this, the high throughout and easy synthesis of quinoxaline have both academic and industrial significances.

In recent years, microdroplet chemistry has emerged to be a facile platform for various types of chemical reactions (Yan et al., 2016, 2018). Micron-sized liquid droplets exhibit several unusual but distinguished reaction features that are not realized in bulk solution (Wei et al., 2020). Especially, the microdroplets created by spray-based ionization/aerosols provide an ultra-rapid mixture of reagents and enable a precise control of reaction conditions including temperature, concentration, and pressure (Girod et al., 2011). Combined with mass spectrometry (MS), the microdroplet can be quickly drawn into the heated inlet to release ionized components, including reaction intermediates and final products, which facilities the simultaneous monitoring of the reaction process. To perform such kind of experiments, several MS instruments have been employed (Yan et al., 2016). The milestone work was conducted in 2011 by Girod et al. who adopted the desorption electrospray ionization (DESI) for the generation of microdroplets (Girod et al., 2011). Besides, electrospray ionization (ESI) was also used for performing organic reactions (Alam et al., 2010). To date, ESI/electrosonic spray ionization (ESSI), (Müller et al., 2012; Banerjee and Zare, 2015) ESSI/microdroplet fusion, (Lee et al., 2015) nanoESI, (Li et al., 2016), and paper spray (Yan et al., 2013) have been demonstrated to be effective platforms for microfluidic-assisted syntheses. Benefitting from the unique conditions in the microdroplet, the chemical reactions demonstrated several distinct features that are not realized in the solution phase: (1) new reaction pathways and mechanisms have been observed for some complex reactions such as Biginelli reaction, Pomeranz-Fritsch synthesis of isoquinoline, and organometallic reactions (Santos et al., 2007; Yan et al., 2014; Lee et al., 2015; Sahota et al., 2019) (2) A considerably accelerated reaction speed can be realized. For instance, Cooks's group has reported an extraordinary rate-enhanced Claisen–Schmidt condensation under base-catalyzed sprayed droplet conditions. The whole reaction process can be accomplished within 2.5 min, which was much faster than the bulk reactions (usually needs several hours) (Müller et al., 2012). (3) A significantly enhanced reaction yield is achieved. For instance, for the preparation of secondary amides from ketoximes through the well-known Beckmann rearrangement, Liu et al. have employed the microdroplet technique and reached a yield as high as 78.7–91.3% for benzoylaniline within several seconds, compared to a yield of typically 10.1–66.1% in several hours in the bulk phase (Zhang et al., 2019).

**Figure 1:**
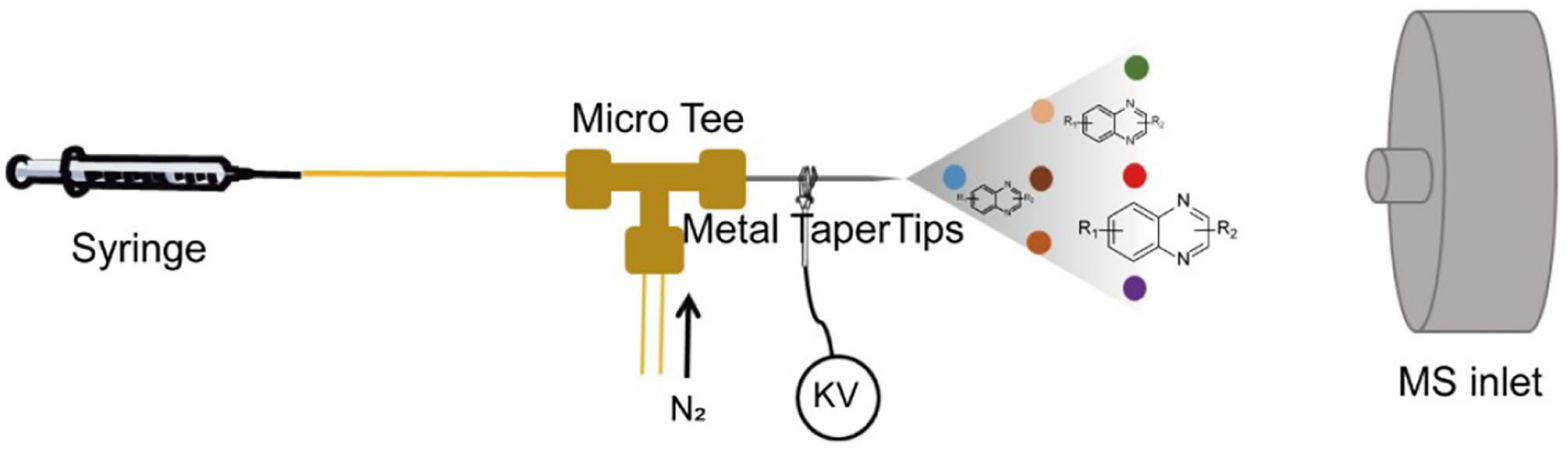
The single-barrel microdroplet device for the synthesis of quinoxaline.

Intrigued by these distinct features of the microdroplet-assisted reactions, here we report the use of a microdroplet reaction for the rapid and high-throughput screening of optimized conditions of the condensation reaction, which has been successfully utilized for the synthesis of substituted quinoxalines. As illustrated in Figure 1, the high-speed stream of the mixture solution, containing two starting materials, was generated by the nebulization of the turbulent nitrogen gas. Then, the condensation reaction was initiated by the rapid mixture of the charged droplet sprayed by the high voltage. To screen the optimized reaction condition, several impacts of droplet volume, reaction flow rate, distance from the MS inlet, spray voltage, and flow rate of the auxiliary gas N_2_ have been systematically studied. Our results clearly demonstrated that the reaction could be completed in microseconds and the conversion rate reached 90% without adding any catalyst. Furthermore, this platform can also be used for high-concentration microdroplet synthesis of quinoxaline diversities. We believe that this simple, fast, and efficient method can be extended for screening the optimized synthesis route of multi-types of substances that need long reaction times or complicating the synthesizing procedure.

## Materials and Methods

### Materials and Instruments

2,3-Diaminotoluene, 3-nitro-1,2-phenylenediamine, naphthalene-2,3-diamine, bibenzyl, 1-phenyl-1, 2-propanedione, and 1,2-indandione were purchased from Hiens Biochemical Technology Co., Ltd. (Tianjin, China). Benzene-1,2-diamine was purchased from Macklin Biochemical Co. Ltd (Shanghai, China). 4-Hydroxyphenylglyoxal hydrate was from J&K Scientific Ltd. (Beijing, China). HPLC-grade methanol was purchased from Fisher. Sartorius ultrapure water (18.2 MΩ cm) was purified with a Sartorius Arium 611 VF system (Sartorius AG, Germany). Unless otherwise stated, all chemicals were used without further purification.

Metal TaperTips and Fittings Kit were purchased from New Objective, Inc. (U.S.). A C18 column (3 μm, 2.1 mm i.d. × 150 mm) was utilized for HPLC analysis on an Ultimate 3000 LC system with 0.3 mL·min^−1^ of methanol/water containing 0.1% formic acid as mobile phase. Mass spectra (MS) were recorded using an LCQ Fleet mass spectrometer (Thermo Fisher Scientific). Absorption and fluorescence spectra were recorded on a UV-2600 UV–visible spectrophotometer (Shimadzu, Japan) and FLS-920 Edinburgh fluorescence spectrometer, respectively. ^1^H NMR spectra were recorded by Bruker Avance 400 MHz spectrometers (Bruker, Germany).

### Synthesis of Quinoxalines With Microdroplet Reaction

The 1,2-diamine and 1,2-dicarbonyl compounds were dissolved in methanol–water solution (v: v = 1: 1), respectively. The two solutions were mixed together in equal proportion and loaded into a microsyringe, followed by injection into the single-barrel microdroplet device through a syringe pump. Then, a high voltage was applied to the Metal TaperTips and N_2_ was used as an auxiliary gas.

### Online Monitoring of Microdroplet Reactions by MS

An LCQ Fleet mass spectrometer (Thermo Fisher Scientific) was used to monitor the reaction processes. The single-barrel microdroplet spray device for generating droplets was placed at 8 mm from the MS inlet capillary, and a high voltage was applied to the Metal TaperTips. For convenience, a 3D-printed box was used to fix this device (Supplementary Figure 1). Due to the high sensitivity of MS, the concentration of the reactants (diamine and 2-dicarbonyl compound) was 0.1 mM. The MS experiments were conducted in positive mode operated under the following conditions: capillary temperature was 350°C; capillary voltage was 10 V; and tube lens was 90 V. In order to obtain the maximum abundance of products, several other parameters have been tested and adjusted, such as the distance between the spray source and the MS injection, the spray voltage, and the solution flow rate.

### Offline Monitoring of Microdroplet Reactions by LC-MS

1,2-Diamine (1 mM) and 1,2-dicarbonyl compounds (1 mM) were dissolved in methanol–water solution (v: v = 1: 1), respectively. The two solutions were mixed together in equal proportion and loaded into a microsyringe. Then, the mixture was injected into the single-barrel microdroplet device through a syringe pump at a flow rate of 3 μL/min. The middle end of the MicroTee was added with 40 psi of N_2_ as an auxiliary gas, and the Metal TaperTips was applied with a voltage of 3.8 kV. The experiments were conducted at room temperature.

The spray was finally collected into a glass bottle. Five microliter of the solution was taken and diluted with 80 μL solvent (methanol: water = 1: 1). The components of the diluted solution were monitored by LC-MS with UV at 254 nm on an Ultimate 3000 LC system coupled with an LCQ Fleet mass spectrometer. A C18 column (3 μm, 2.1 mm i.d. × 150 mm) was utilized for LC analysis with methanol/water with 0.1% formic acid as mobile phase, and the eluent flow rate was 0.3 ml/min. The MS operated in positive mode using the following parameters: full-scan mass spectra were acquired over the m/z range from 50 to 1,000 using LCQ Fleet. The monitoring was conducted under the following conditions: sheath gas flow rate was 30 arb; aux gas flow rate was 10 arb; spray voltage was 3.8 kV; capillary temperature was 350°C; capillary voltage was 10 V; and tube lens was 90 V.

### Microdroplet Reaction at High Concentrations

The 1,2-diamine (0.1 M) and 1,2-dicarbonyl compounds (0.1 M) were firstly dissolved in methanol–water solution (v: v = 1: 1). The two solutions were then mixed together in equal proportions and filled into a microsyringe. The mixture was then injected into the single-drop microdropper device through a syringe pump at a flow rate of 3 μL/min. 40 psi of N_2_ was added to the middle end of MicroTee as auxiliary gas, and a voltage of 3.8 kV was applied to the metal TaperTips. All experiments were conducted at room temperature. Then, 5 μl of the reaction solution was taken and mixed with an equal concentration of internal standard. The mixed solution was diluted with 80 μl of methanol–water solution (v: v = 1: 1). The reaction yield was calculated by the chromatogram of LC-MS.

## Results and Discussions

### Microdroplet System and Optimization of Quinoxaline Synthesis Conditions

As shown in Figure 1, this single-barrel microdroplet system involves three parts: (1) a microinjection pump combined with a syringe and capillary for accurate control of the flow rate, (2) a metal taper tip connected with a high voltage for charged microdroplet spray, and (3) an N_2_ gas capillary tube to assist the charged microdroplet movement. A MicroTee connector was used to combine these three parts, which was fixed in a 3D printing box (Supplementary Figure 1).

**Figure 2:**
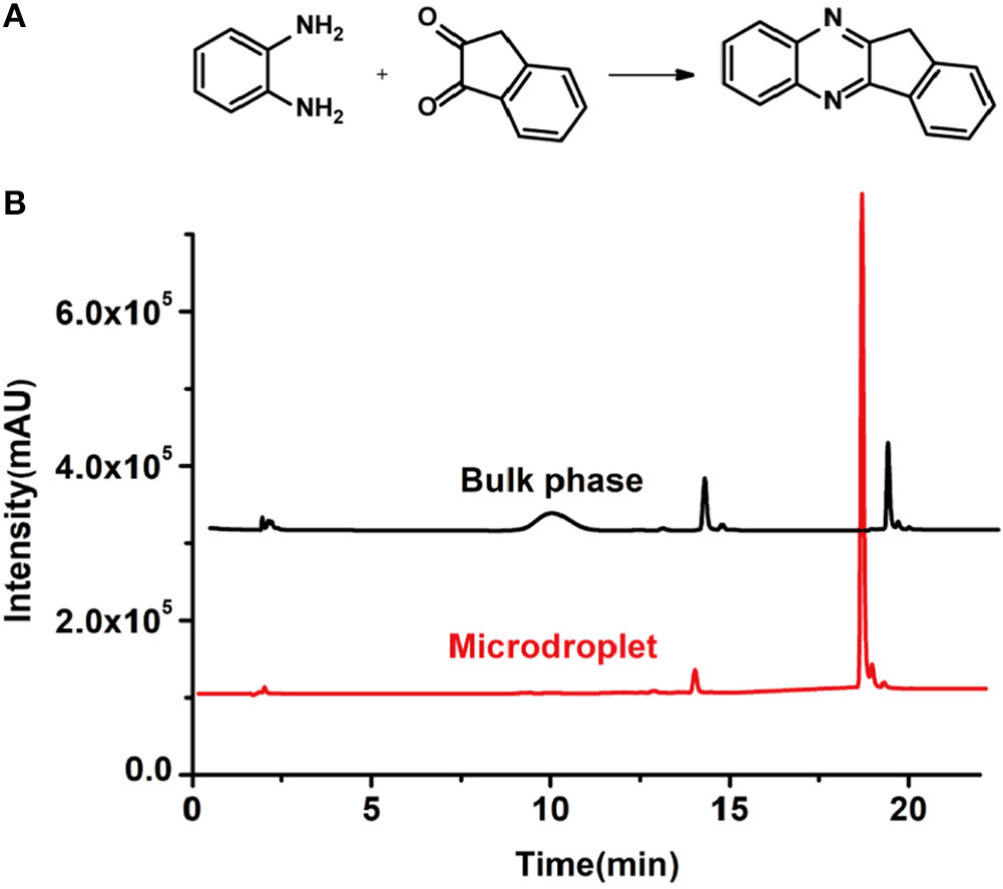
**(A)** The model reaction of benzene-1,2-diamine and 1,2-indanedione. **(B)** Chromatogram of LC-MS for the model reaction under the optimal microdroplet condition (red line) and bulk phase (black line). The retention time is uniformly 18.6 min.

In order to search for the optimized conditions for microdroplet quinoxaline synthesis, a model reaction of benzene-1, 2-diamine, and 1,2-indanedione was chosen (Figure 2A). It is well-known that the charged microdroplet spray generation is affected by several factors, such as voltage, distance between the needle and the inlet of the mass spectrometer, and flow rate. Based on this, the optimization of experimental conditions was performed by using an internal standard (IS, 3,4-dimethylquinoxaline, m/z 159) for MS signal detection. According to the preliminary results, as depicted in Supplementary Figures 2, 3 in the Supporting Information (SI), the high voltage of 3.8 kV was applied to the metal taper tip, which could effectively spray a large amount of charged microdroplets and exhibited a high stability for the product spray ionization. Additionally, it was found that under the optimized conditions of a distance of 8 mm, 40 psi of nebulizing gas (N_2_), and 3 μL/min flow rate of the reactant solution mixture, the highest MS signal ratio of quinoxaline and IS (219/159) was obtained. Under these conditions, the reaction solution of the model reactions of benzene-1,2-diamine and 1,2-indanedione were collected by a glass bottle. Then, 5 μl of the reaction solution was diluted with 80 μL solvent (methanol: water = 1: 1) and further analyzed by the chromatogram of LC-MS.

As a control, the bulk-phase reaction was also conducted. In detail, equal volumes of 1,2-diamine (1 mM) and 1,2-dicarbonyl compound (1 mM) in methanol–water solutions (v: v = 1:1) were mixed and stirred for 10 min under room temperature. Then, 5 μl of reaction solution was taken and diluted with 80 μL solvent for LC-MS analysis. As shown in Figure 2B, it was found that the yield of microdroplet reaction was orders of magnitude higher than the conventional bulk-phase approach, which clearly revealed the distinguished features of both quick and high efficiency of this microdroplet approach.

### Mechanistic Insights to Accelerated Reaction Rate Compared to Bulk Phase

**Figure 3:**
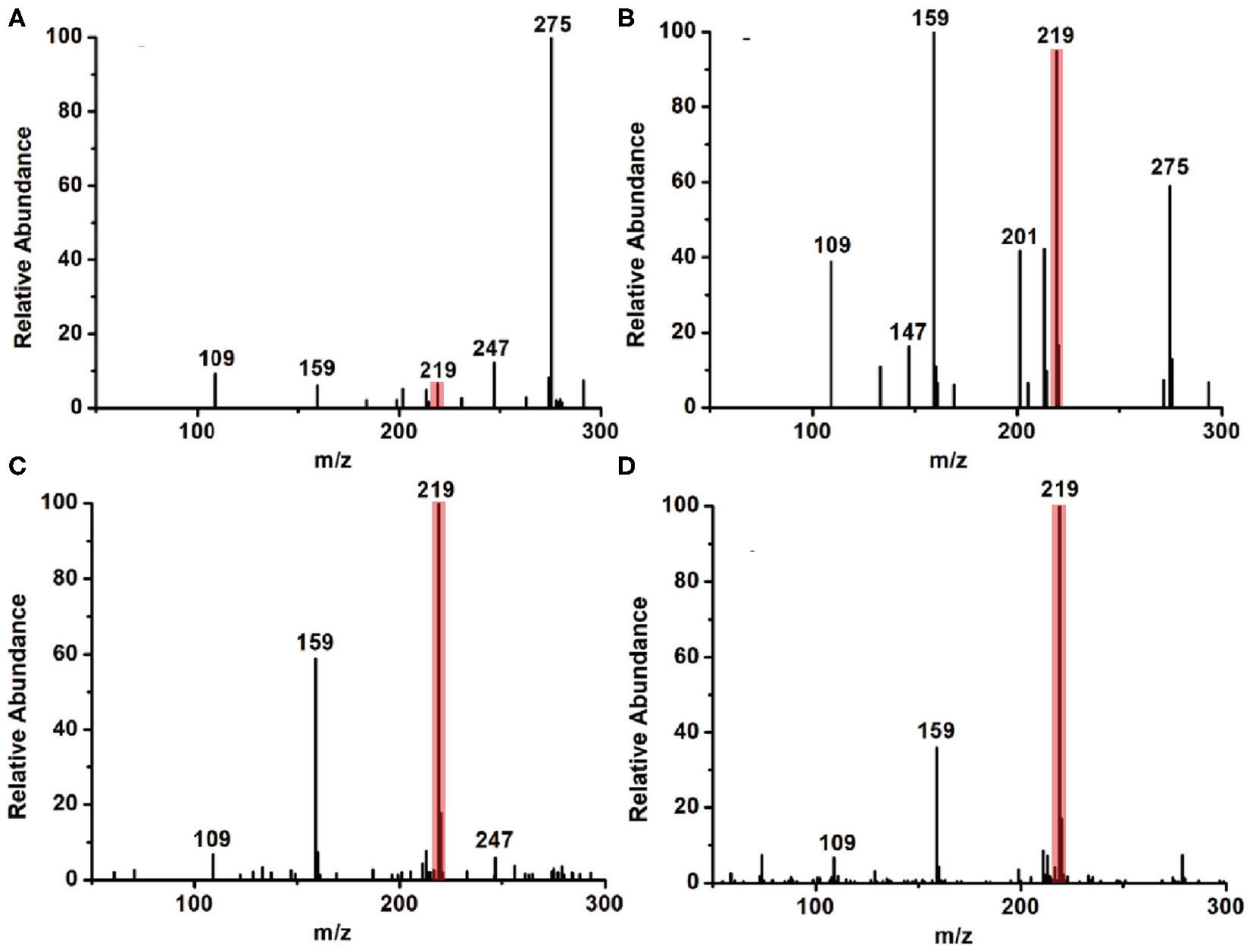
The MS results of the model reaction under **(A)** methanol and **(B)** methanol–water solution (v: v = 1: 1) environment without N_2_. **(C)** The MS results of the model reaction under methanol–water solution (v: v = 1: 1) conditions without N_2_. **(D)** The MS results of the model reaction under methanol–water solution (v: v = 1: 1) conditions with N_2_ (40 psi).

As is well-known, the basis of 1,2-diamines with 1,2-dicarbonyl compounds is a nucleophilic addition elimination reaction; thus, a weak acidic environment is beneficial for this kind of reaction. On the other side, the microdroplet evaporation and the high surface-to-volume ratio of the microdroplet may also contribute to the reaction acceleration. Based on this information, two factors, (1) the ratio of water in reaction solvent and (2) auxiliary gas, were systematically explored to address the mechanism of how the microdroplet approach accelerates the reaction rate. Firstly, we tested two kinds of reaction solvents: methanol only and methanol–water solution (v: v = 1: 1). The microdroplet reaction results are summarized in Figures 3A,B, from which it is seen that the MS signal ratio of the product (11H-indeno[1,2-b]quinoxaline, m/z 219) to the IS (2,3-dimethylquinoxaline, m/z 159) showed a significant increase as the addition of the proportion of water. On the other hand, the MS signal of the raw material (benzene-1,2-diamine, m/z 109) was reduced. This interesting phenomenon was mainly attributed to the introduction of water molecules to the reaction, resulting in an acidic environment under spray phase, which was beneficial for such kind of reaction.

In addition, the size of the generated macrodroplets is another key factor that affects the reaction rate. The coaxial sheath gas was applied to improve the atomization efficiency and guide the generated spray toward the MS inlet. As shown in Figures 3C,D, along with the increase of flow rate of nitrogen gas, the MS signal representing the product (m/z 219) demonstrated a significant increase. In contrast, the MS signal ratio of the raw material (m/z 109) and IS (m/z 159) was found to decrease accordingly. These results reveal that the coaxial sheath gas can significantly increase the microdroplet reaction efficiency. The main reason is that N_2_ gas promotes the evaporation of the reaction solvent, which facilitates the mixing of the reactants, which greatly improved the reaction speed and efficiency.

### Synthesis of Quinoxaline Derivatives in Microdroplets

**Figure 4:**
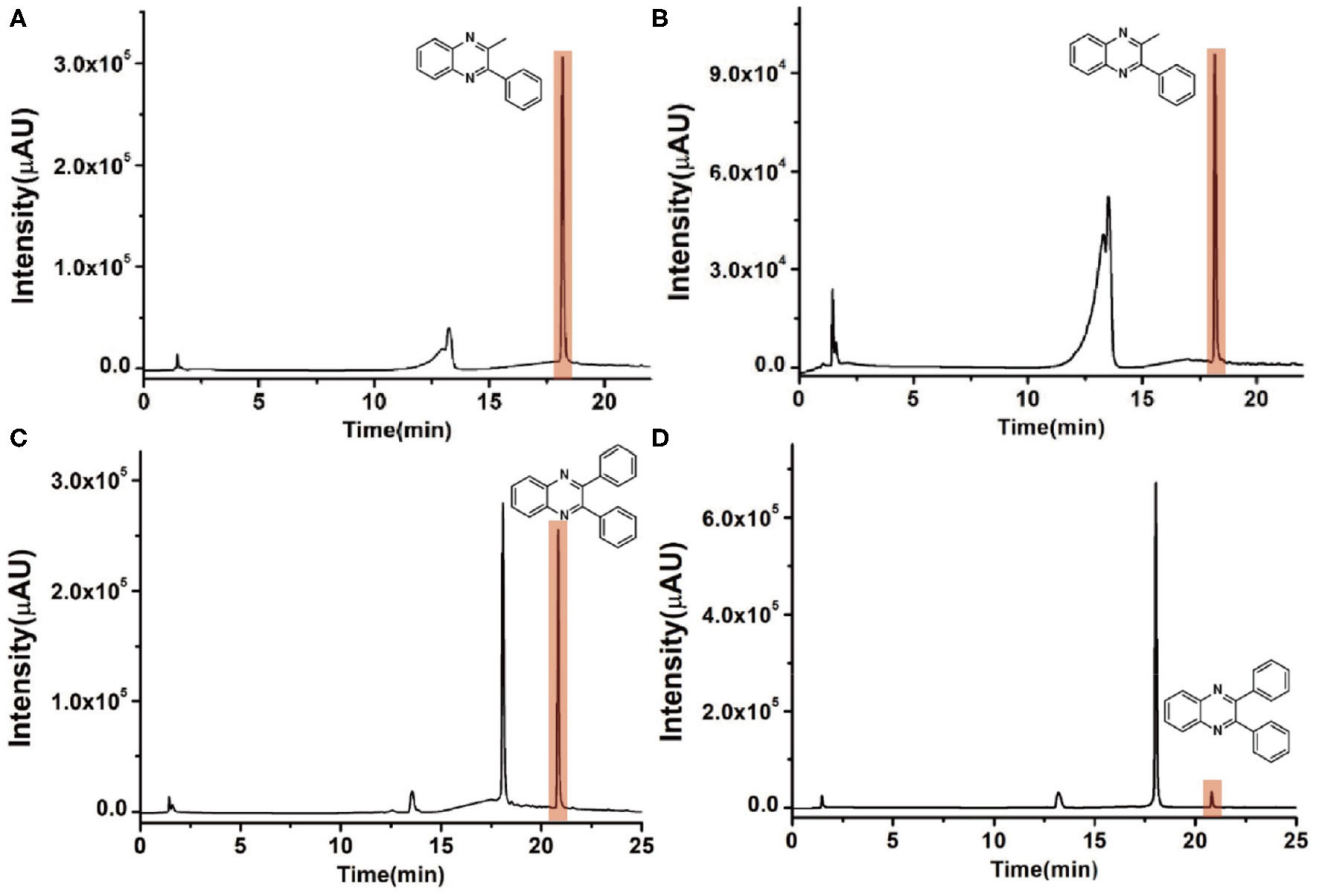
Chromatographic results of LC-MS for the reaction of **(A)** benzene-1,2-diamine and 1-phenyl-1,2-propanedione under microdroplet conditions. **(B)** benzene-1,2-diamine and 1-phenyl-1,2-propanedione in bulk solution. **(C)** Benzene-1,2-diamine and bibenzyl under microdroplet conditions. **(D)** Benzene-1,2-diamine and bibenzyl in bulk solution.

To check the performance of this microdroplet platform, we further extended the condensation reaction to benzene-1,2-diamine with different 1,2-dicarbonyl compounds (as shown in Tables 1, 2) following the same procedure in the aforementioned experiment. All the products were collected and analyzed quantitatively by the chromatogram of LC-MS to calculate the reaction yield. As a comparison, the corresponding bulk-phase reaction was also performed under the same conditions. As shown in Figures 2B, 4A,C, for the three quinoxaline derivatives under examination, a large amount of quinoxaline products was successfully formed under microdroplet conditions and almost no signals from the reactants were detected. In contrast, the yields in the bulk-phase reaction were considerably lower than that of the microdroplet reaction. Meanwhile, there was still plenty of benzene-1,2-diamine and 1,2-dicarbonyl reactants left in the bulk-phase reaction (Figures 2B, 4B,D). This phenomenon solidly reveals that microdroplets reaction can complete in a much faster and more efficient manner than the bulk-phase reaction. By quantitatively calculating the reaction conversion efficiency of three cases as summarized in Table 1, it is clear that all of the reaction conversion efficiency based on microdroplet method was significantly higher than that of the solution phase, revealing the distinguished performance of the microdroplet platform in this kind of reaction. To further confirm the product structure, all of the products were collected and characterized by NMR (Supplementary Figures 5–7).

**Table 1:** Synthesis of various quinoxalines in microdroplets and bulk solution.

**Reactant 1**	**Reactant 2**	**Product**	**Yield^a^**	**Yield^b^**
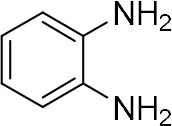	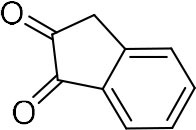	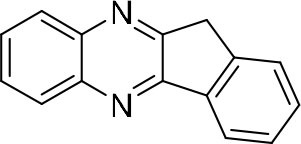	31.6%	98.5%
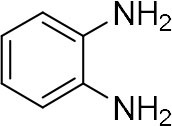	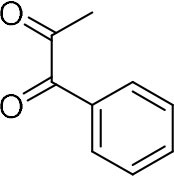	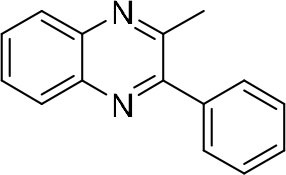	25.58%	82.76%
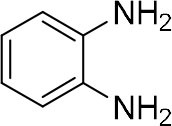	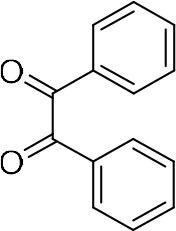	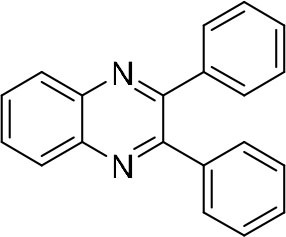	4.65%	43.88%

*Yield^a^ is the reaction yield in the solution phase for 10 min*.

*Yield^b^ is the reaction yield under microdroplet conditions*.

**Table 2:** Synthesis of various quinoxalines in microdroplets and bulk solution.

**Reactant 1**	**Reactant 2**	**Product**	**Yield^a^**	**Yield^b^**
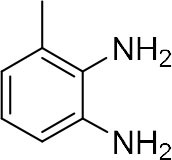	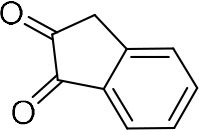	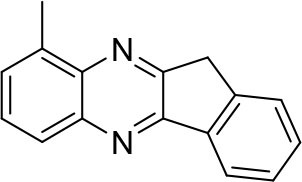	13.05%	70.23%
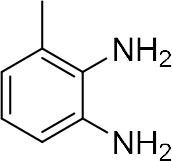	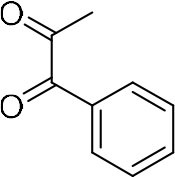	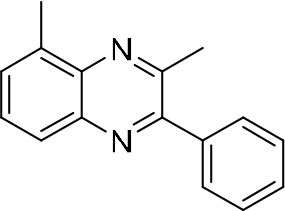	21.58%	90.73%
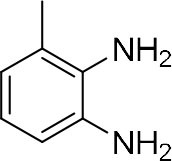	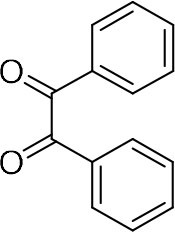	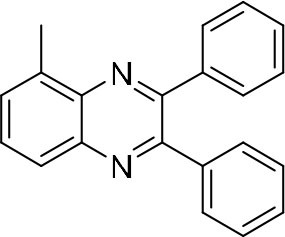	3.35%	9.00%
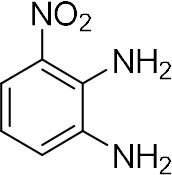	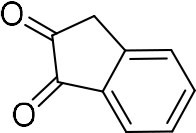	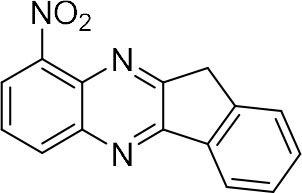	8.2%	13.39%
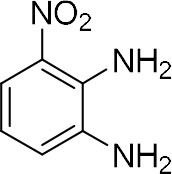	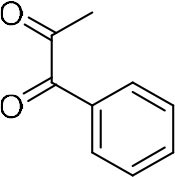	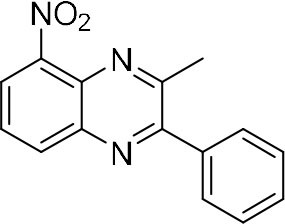	20.76%	54.20%

*Yield^a^ is the reaction yield in the solution phase for 10 min*.

*Yield^b^ is the reaction yield under microdroplet conditions*.

### Single-Barrel Microdroplet for *o*-Sub Quinoxaline Derivatives Synthesis

In the following, we further tried the reaction of benzene-1,2-diamine with electron-donating and electron-withdrawing groups at the ortho position to different diones. To accelerate the reaction (Meshram et al., 2010), 0.1% formic acid was applied. Taking 2,3-diaminotoluene and 1,2-indanedione for example, the yield under the microdroplet condition reached 70.23%, compared to a yield of only 13.05% under the bulk phase. All of the products were collected and quantitatively analyzed by the chromatogram of LC-MS to calculate the efficiency. As shown in Table 2, the yield under microdroplet conditions is also found to be significantly higher than the bulk phase. To further confirm the product structure, the crude products were collected and characterized by LC-MS and NMR (Supplementary Figures 4, 8–12).

### Single-Barrel Microdroplet for Direct Quinoxaline Dye Synthesis

**Figure 5:**
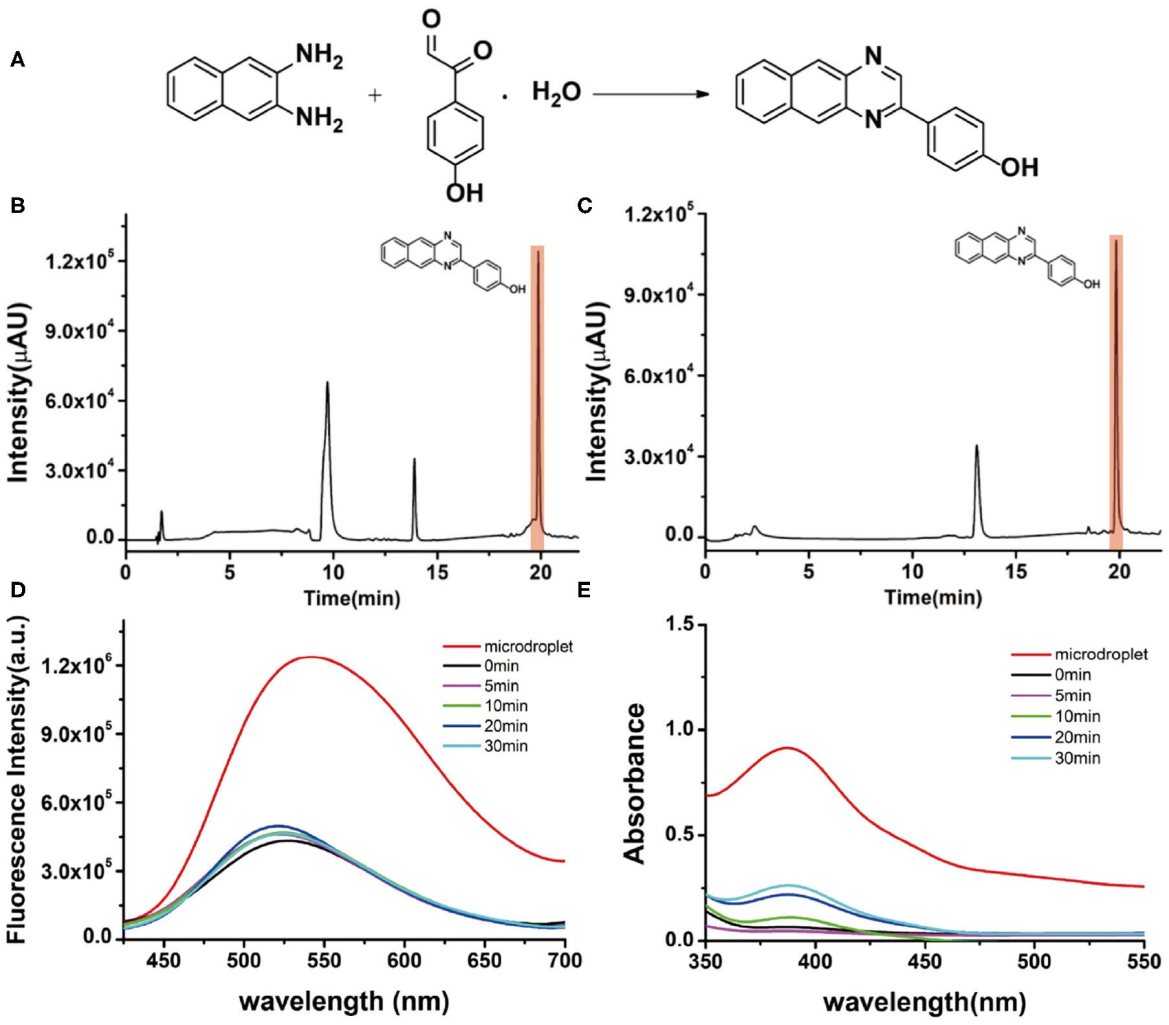
**(A)** The synthesis of the fluorescent viscosity probe. Chromatographic results of LC-MS for the products **(B)** in bulk-phase stirring for 10 min and **(C)** in the microdroplet. **(D)** Fluorescence emission spectra of the products of the microdroplet reaction and bulk reaction at different reaction times (0, 5, 10, 20, 30 min). **(E)** UV/visible spectra of microdroplet reactions and bulk reaction at different reaction times (0, 5, 10, 20, 30 min).

Viscosity is a basic physicochemical factor that affects physiological processes (Wang et al., 2013). Several diseases are caused by the alteration of viscosity in the biological environment, such as cholesterolemia, atherosclerosis, cellular malignancies, and diabetes (Yang et al., 2020). Up to now, many viscosity-sensitive fluorescent probes have been developed and successfully utilized to monitor the viscosity change in proteins or cell membranes (Kuimova, 2012; Bui et al., 2017). Therefore, the synthesis of viscosity-sensitive fluorescent probes is clinically desirable but typically requires a complicated synthesis procedure. Considering this, we also tested our single-barrel microdroplet device to synthesize a model fluorescent viscosity probe, 4-(benzo[g]quinoxalin-2-yl)phenol. Naphthalene-2,3-diamine and 2-(4-hydroxyphenyl)-2-oxoacet-aldehyde were chosen as reagents (Figure 5A). The synthesis process was the same as mentioned above. The product solution was collected and quantitatively analyzed by the chromatogram of LC-MS, fluorescence emission spectra, and UV/visible spectroscopy. For a bulk solution reaction as shown in Figure 5B, the clear peak at a retention time of 10 min indicated that a large amount of reactant of naphthalene-2,3-diamine still exists, revealing the low efficiency in the solution phase. In contrast, the peak of the starting material naphthalene-2,3-diamine in the microdroplet reaction almost disappeared, which indicated the complete reaction process (Figure 5C). From the UV/Vis spectroscopic spectrum (Figure 5D), it can be seen that the intensity of the absorption band at 390 nm is considerably higher for microdroplet reaction than bulk solution reaction, indicating the success formation of the fluorescent probe in microdroplets. This is further confirmed by the fluorescence emission spectrum shown in Figure 5E, because the microdroplet reaction has a higher fluorescence intensity at 530 nm than the bulk solution phase.

## Conclusions

In this work, the microdroplet technique has been utilized for reaction condition screening to search for the optimized conditions of the synthesis of quinoxaline derivatives. Compared with the bulk-phase reaction, the yields of quinoxalines in microdroplets were improved significantly, and the reaction time was greatly shortened to be within several seconds. From our results, methanol–water solvent (v: v = 1:1) offered the best conversion efficiency of products. Online MS detection indicated that the formation of products can be completed within a microsecond timescale in microdroplets. Offline quantitative characterization of the product further proves the higher efficiency of microdroplet reaction than the bulk phase. The present platform offers several unique advantages, including shorter reaction time, simple operation, significantly enhanced yields, and a greener aspect by avoiding the need of the catalyst. The preparative-scale experiment was also performed and yielded a product at an isolated rate of 1.2 mg. min^−1^, which revealed the high potential of the present method.

## Data Availability Statement

All datasets generated for this study are included in the article/Supplementary Material.

## Author Contributions

YY, JL, ZC, and BT devised and designed the study. PY and XM contributed to the reaction design aspects of the study. YY, JL, WN, RL, and LN undertook the experimental work. All authors contributed to writing and editing of the manuscript.

## Conflict of Interest

The authors declare that the research was conducted in the absence of any commercial or financial relationships that could be construed as a potential conflict of interest.
