# Supplementary material for: A High-Throughput Screening Method for Determining the Optimized Synthesis Conditions of Quinoxaline Derivatives Using Microdroplet Reaction
Source: Front Chem. 2020 Sep 10;8:789. doi: 10.3389/fchem.2020.00789 (PMC7533680; doi:10.3389/fchem.2020.00789)
Supplement: Supplementary file 1 [file Image_1.pdf]

Supplementary Material

1 The Setup of the Single Barrel Microdroplet Device

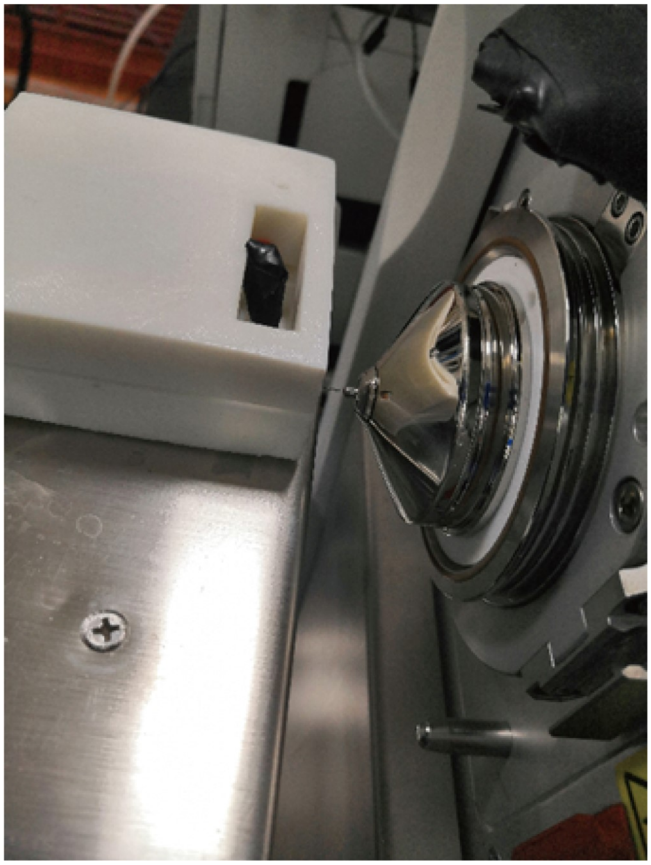

Supplementary Figure 1. Photograph of the microdroplet reactor device

2 Screening of Optimal Conditions for Quinoxaline Synthesis in Single Barrel Microdroplet

The microdroplet droplet spray is affected by factors including spray voltage, flow rate, and the distance between the metal TaperTips and the inlet of the mass spectrometer. Thus, the screening of optimam conditions for reaction was firstly conducted on the bases of the MS signal ratio of quinoxaline and IS (219/159). As shown in Supplementary Figure 2, the spray voltage in a range of 1~4 kV, distance of 3~11 mm, and flow rate from 1 to 9 µL/min were systematically tested.

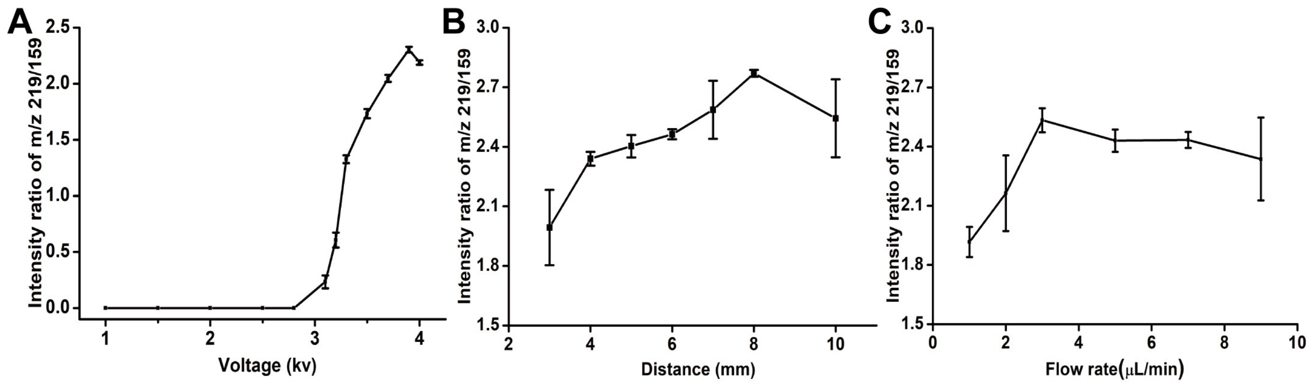

Supplementary Figure 2. Optimization of experimental conditions based on the MS signal ratio of quinoxaline and IS (219/159). **(A)** The spray voltage; **(B)**Horizontal distance between the metal TaperTips and MS inlet; **(C)** Reaction flow rate.

The N_2 flow rate also has a great influence on the peak output, so we screened different N_2 flow rates for the peak output of the reaction. As shown in the figure below, we tested the peak value of N_2 flow at 10-80 psi. In the case of 10-30 psi, the peak of the reaction product 219 is higher, but the internal standard peak 159 is also higher, and the other peaks are very messy. When the nitrogen flow rate is 40 psi, the peak signal of the reaction product peak 219 is the highest, and the internal standard peak 159 is low, and there are no other interference peaks. At 50-80 psi, both reaction product peak 219 and internal standard peak 159 are higher. Therefore, we used nitrogen at a flow rate of 40 psi to assist the reaction.

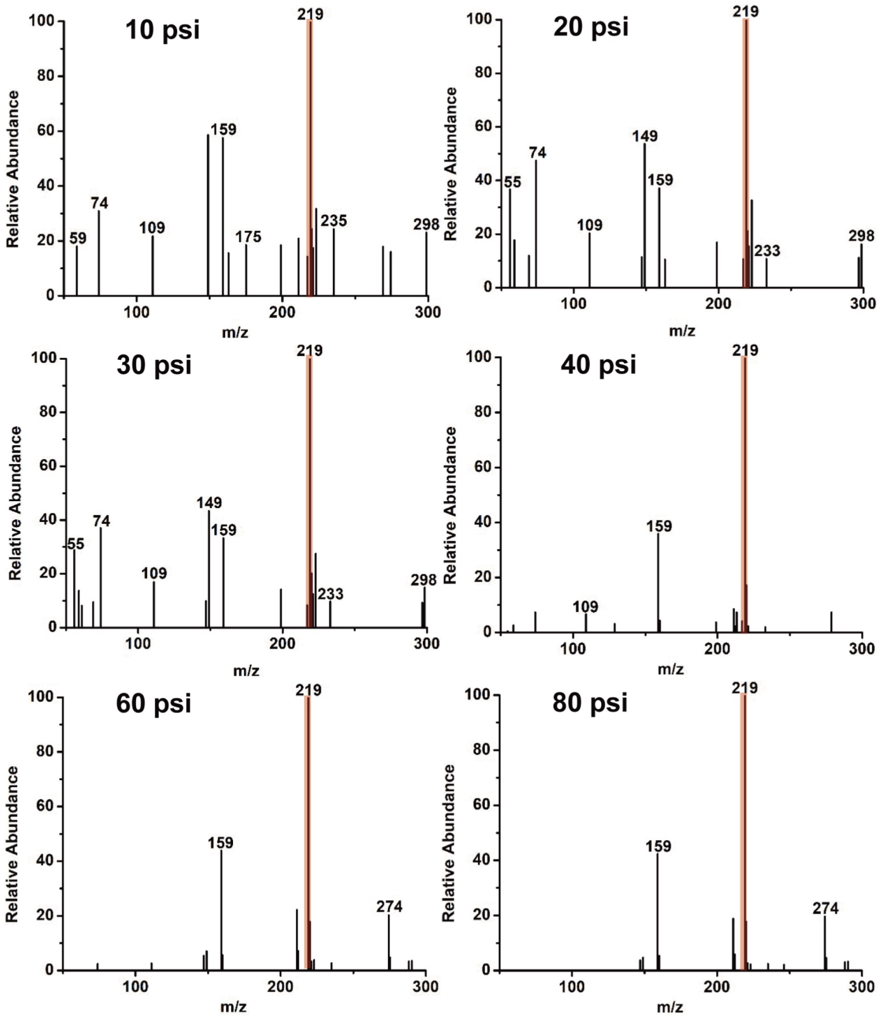

Supplementary Figure 3. The figure shows the peak of the mass spectrum under different flow rates of N_2.

3 The chromatogram of LC-MS Analyses of *o*-Sub Quinoxaline Derivatives

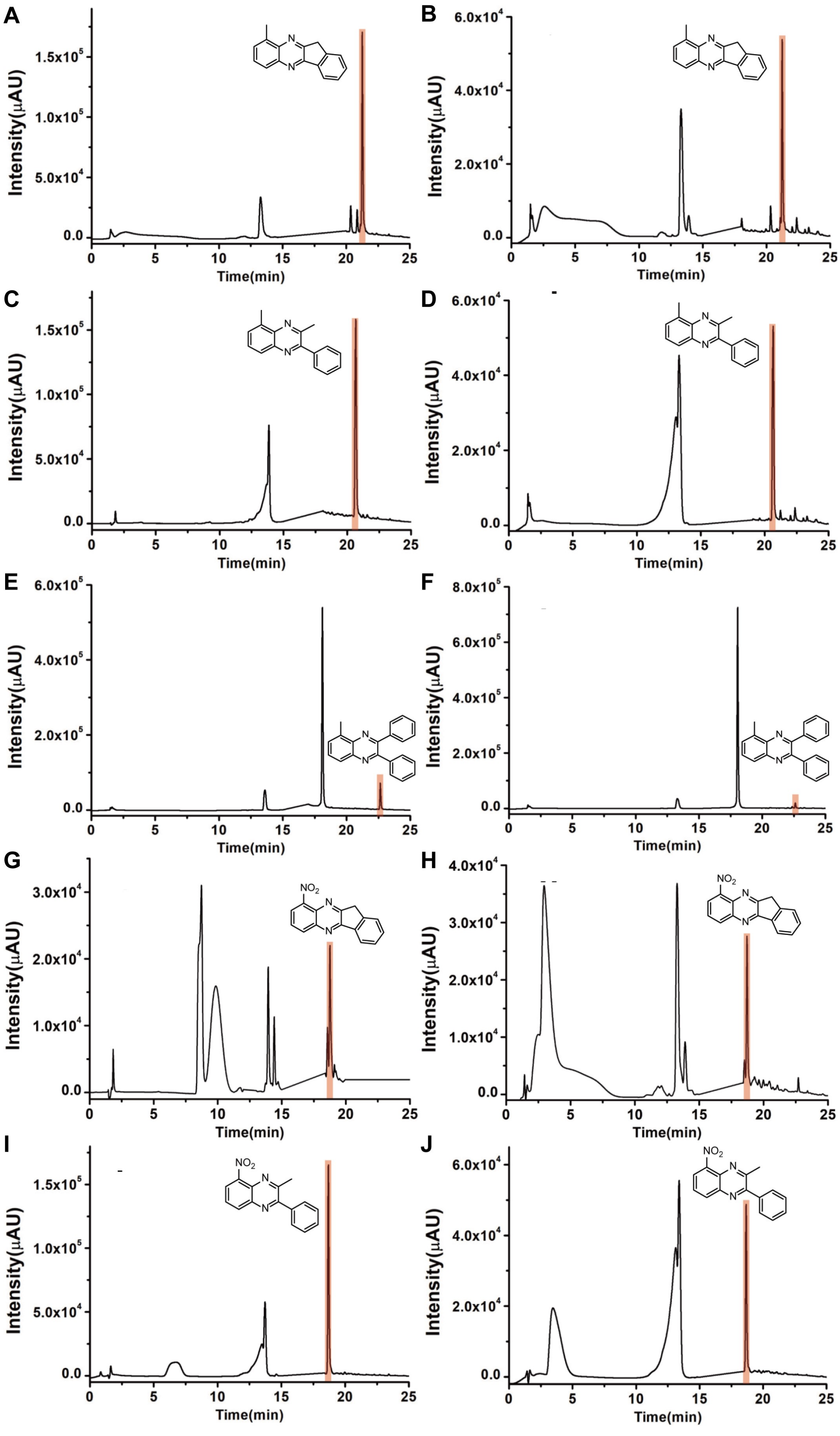
A
B
C
D
E
F
G
H
I
J
Intensity(μAU)
Time(min)
NO2
N
0.0
5.0x10^4
1.0x10^5
1.5x10^5
2.0x10^4
4.0x10^4
6.0x10^4
2.0x10^5
4.0x10^5
6.0x10^5
8.0x10^5
1.0x10^4
3.0x10^4
0
5
10
15
20
25

Supplementary Figure 4. The chromatogram of LC-MS analyses of **(A)** 2, 3-diaminotoluene and 1,2-Indanedione reaction between in microdroplet, **(B)** 2, 3-diaminotoluene and 1,2-Indanedione reaction in the bulk phase, **(C)** 2, 3-diaminotoluene and 1-phenyl-1,2-propanedione in the microdroplet, **(D)** 2, 3-diaminotoluene and 1-phenyl-1,2-propanedione in the bulk phase, **(E)** 2, 3-diaminotoluene and Benzil in the microdroplet, **(F)** 2, 3-diaminotoluene and Benzil in the bulk phase, **(G)** 3-nitro-1,2-phenylenediamine and 1,2-Indanedione in microdroplets, **(H)** 3-nitro-1,2-phenylenediamine and 1,2-Indanedione bulk phase, **(I)** 3-nitro-1,2-phenylenediamine and 1-phenyl-1,2-propanedione in the microdroplet and **(J)** 3-nitro-1,2-phenylenediamine and 1-phenyl-1,2-propanedione in bulk phase, respectively. The orange color highlighted peaks represent the product.

4 Synthetic Steps of Various Quinoxalines in Bulk Phase

For synthesis of 2, 3-dialkyl quinoxalines, benzene-1, 2-diamine (10 mmol) and ammonium chloride (5 mmol) were added to a stirred solution of 2, 3-dione in methanol (13 mmol, 50 mL), which was further stirred for 1 hour at room temperature. Then the mixture was washed with water (200 mL) and extracted by CH_2Cl_2. The organic phase was evaporated under reduced pressure to obtain the pure product.

For synthesis of 2, 3-diphenyl quinoxalines and 2-aryl-3-alkyl quinoxalines, saccharin (0.5 mmol) and 1, 2-arylenediamine (10 mmol) were added to a methanol solution of 1-phenylpropane-1, 2-dione (10 mmol, 10 mL). The mixture was stirred for appropriate time at room temperature, which was monitored by thin layer chromatography, until the reaction was fully completed. Then, the solvent was removed under reduced pressure to collect the crude product which was further purified by flash column chromatography in silica gel (eluent, 90:10 petroleum ether/ethyl acetate) to obtain quinoxalines.

All of the quinoxaline diversities were characterized by ^{1}H NMR and HRMS:

11H-indeno[1,2-b]quinoxaline. Yellow solid, 45.3mg, 90.6% yield**.** ^{1}H NMR (400 MHz, DMSO) δ 8.23 – 8.05 (m, 3H), 7.91 – 7.71 (m, 3H), 7.69 – 7.50 (m, 2H), 4.21 (s, 2H). HRMS (ESI) (m/z): $[M+H]^+$ calculated for $C_{15}H_{10}N_2$, 219.0917; found, 219.0935.

2-methyl-3-phenylquinoxaline. Yellow liquid, 47.6mg, 95.2% yield**.**^{1}H NMR (400 MHz, DMSO) δ 8.05 (s, 2H), 7.83 (t, J = 9.9 Hz, 2H), 7.74 (s, 2H), 7.56 (d, J = 7.2 Hz, 3H), 2.72 (s, 3H). HRMS (ESI) (m/z): $[M+H]^+$ calculated for $C_{15}H_{12}N_2$, 221.1073; found, 221.1058.

2, 3-diphenylquinoxaline. White solid, 43.2mg, 86.4% yield. ^{1}H NMR (400 MHz, DMSO) δ 8.12 (dd, J = 6.4, 3.4 Hz, 2H), 7.84 (dd, J = 6.4, 3.4 Hz, 2H), 7.44 (d, J = 6.3 Hz, 4H), 7.39 – 7.26 (m, 6H). HRMS (ESI) (m/z): $[M+H]^+$ calculated for $C_{20}H_{14}N_2$, 283.1230; found, 283.1269.

9-methyl-11H-indeno[1,2-b]quinoxaline. Yellow solid, 35.2mg,70.4% yield.^{1}H NMR (400 MHz, DMSO) δ 8.09 (d, J = 7.0 Hz, 1H), 7.88 – 7.81 (m, 1H), 7.68 (t, J = 9.1 Hz, 1H), 7.58 – 7.45 (m, 4H), 4.12 (s, 1H), 2.75 (s, 1H). HRMS (ESI) (m/z): $[M+H]^+$ calculated for $C_{16}H_{12}N_2$, 233.1073; found, 233.1032.

3, 5-dimethyl-2-phenylquinoxaline. White solid, 37.8mg, 75.6% yield.^{1}H NMR (400 MHz, DMSO) δ 7.86 (t, J = 13.8 Hz, 1H), 7.80 – 7.63 (m, 3H), 7.61 – 7.40 (m, 2H), 2.72 (s, 2H), 2.71 (s, 2H). HRMS (ESI) (m/z): $[M+H]^+$ calculated for $C_{16}H_{14}N_2$, 235.1230; found, 235.1244.

5-methyl-2, 3-diphenylquinoxaline. White solid, 33.9mg, 67.8% yield.^{1}H NMR (400 MHz, DMSO) δ 8.04 – 7.86 (m, 2H), 7.86 – 7.67 (m, 2H), 7.68 – 7.58 (m, 1H), 7.57 – 7.44 (m, 3H), 7.44 – 7.27 (m, 5H), 2.78 (s, 3H). HRMS (ESI) (m/z): $[M+H]^+$ calculated for $C_{21}H_{16}N_2$, 297.1386; found, 297.1345.

9-nitro-11H-indeno[1,2-b]quinoxaline. Red solid, 32.4mg, 64.8% yield.^{1}H NMR (400 MHz, DMSO) δ 8.44 (d, J = 8.4 Hz, 1H), 8.31 (d, J = 7.6 Hz, 1H), 8.21 (d, J = 7.5 Hz, 1H), 7.97 (t, J = 8.0 Hz, 1H),

7.81 (d, J = 7.6 Hz, 1H), 7.69 (t, J = 6.9 Hz, 1H), 7.62 (t, J = 7.4 Hz, 1H), 4.29 (s, 2H). HRMS (ESI) (m/z): $[M+H]^+$ calculated for $C_{15}H_9N_3O_2$, 264.0768; found, 264.0732.

3-methyl-5-nitro-2-phenylquinoxaline. Yellow solid, 39.6mg, 79.2% yield. ^1H NMR (400 MHz, DMSO) δ 8.36 (d, J = 7.6 Hz, 2H), 7.95 (t, J = 8.0 Hz, 1H), 7.84 – 7.72 (m, 2H), 7.65 – 7.51 (m, 3H), 2.73 (s, 3H). HRMS (ESI) (m/z): $[M+H]^+$ calculated for $C_{15}H_{11}N_3O_2$, 266.0924; found, 266.0936.

4-(benzo[g]quinoxalin-2-yl)phenol. Yellow solid，44.5mg, 89% yield. ^1H NMR (400 MHz, DMSO) δ 10.17 (s, 1H), 9.58 (s, 1H), 8.69 (dd, J = 26.7, 9.4 Hz, 2H), 8.46 – 8.10 (m, 4H), 7.79 – 7.51 (m, 2H), 7.00 (d, J = 8.7 Hz, 2H). HRMS (ESI) (m/z): $[M+H]^+$ calculated for $C_{18}H_{12}N_2O$, 273.1022; found, 273.1042.

5 ^1H NMR Spectra of Various Quinoxalines

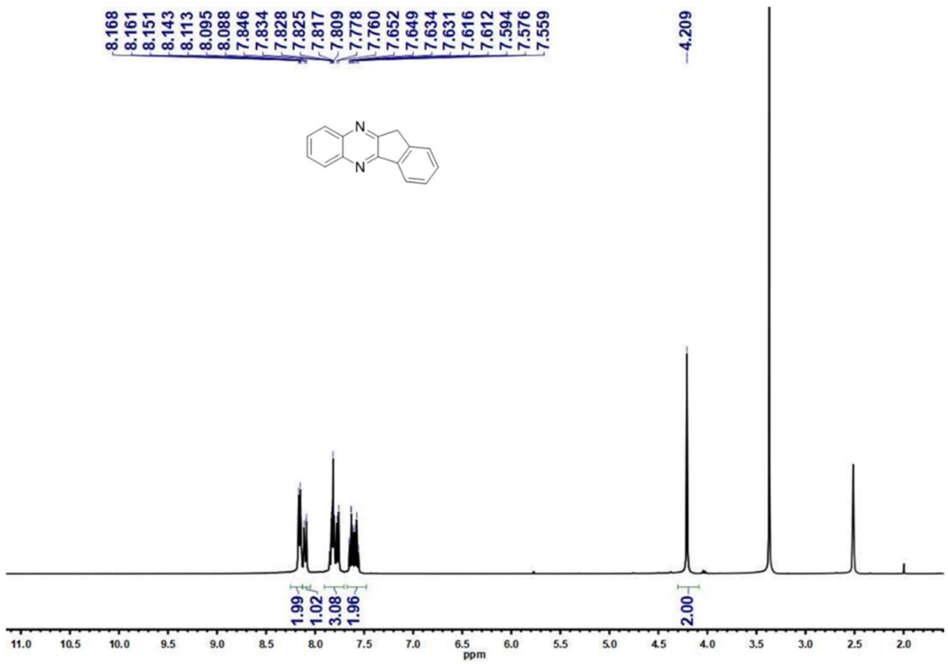

Supplementary Figure 5. ^{1}H NMR spectrum of 11H-indeno[1,2-b]quinoxaline

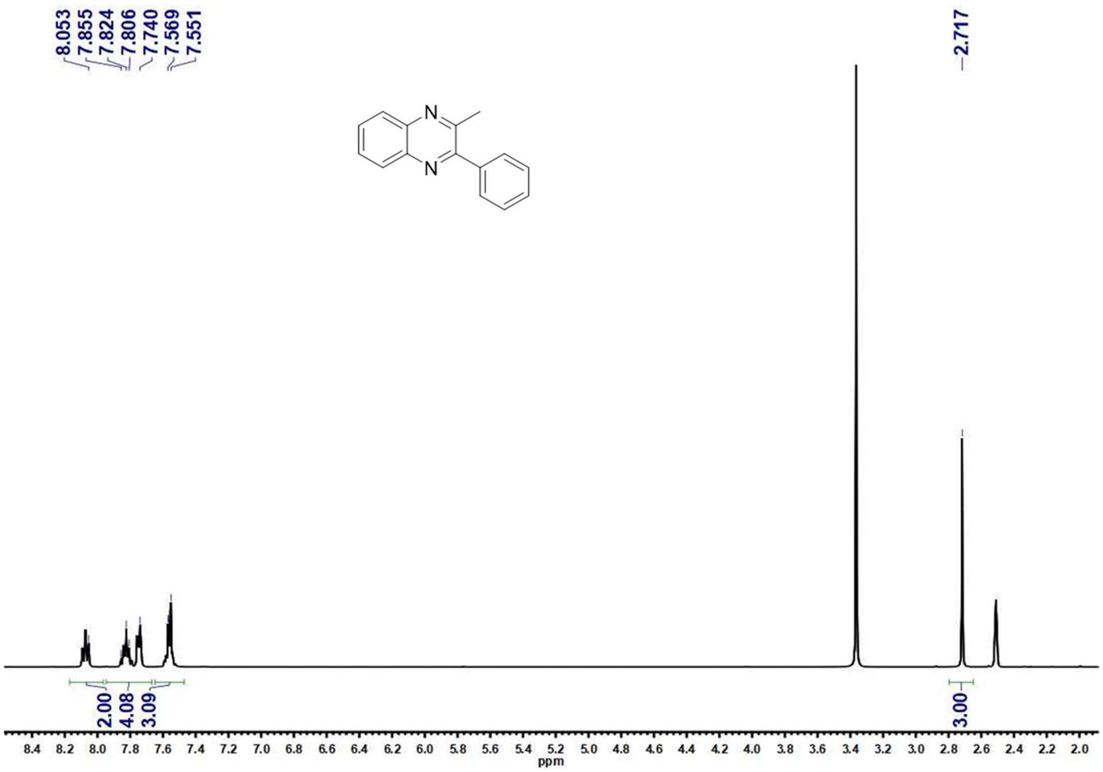

Supplementary Figure 6. ^{1}H NMR spectrum of 2-methyl-3-phenylquinoxaline.

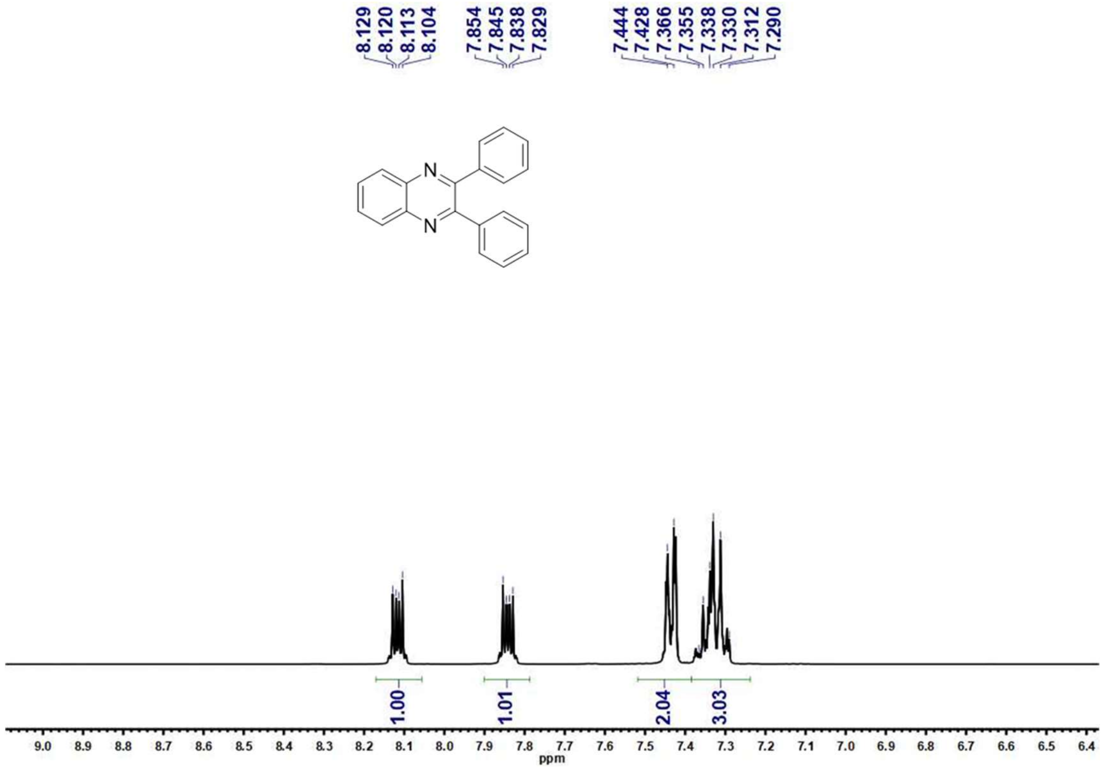

Supplementary Figure 7. ^{1}H NMR spectrum of 2, 3-diphenylquinoxaline.

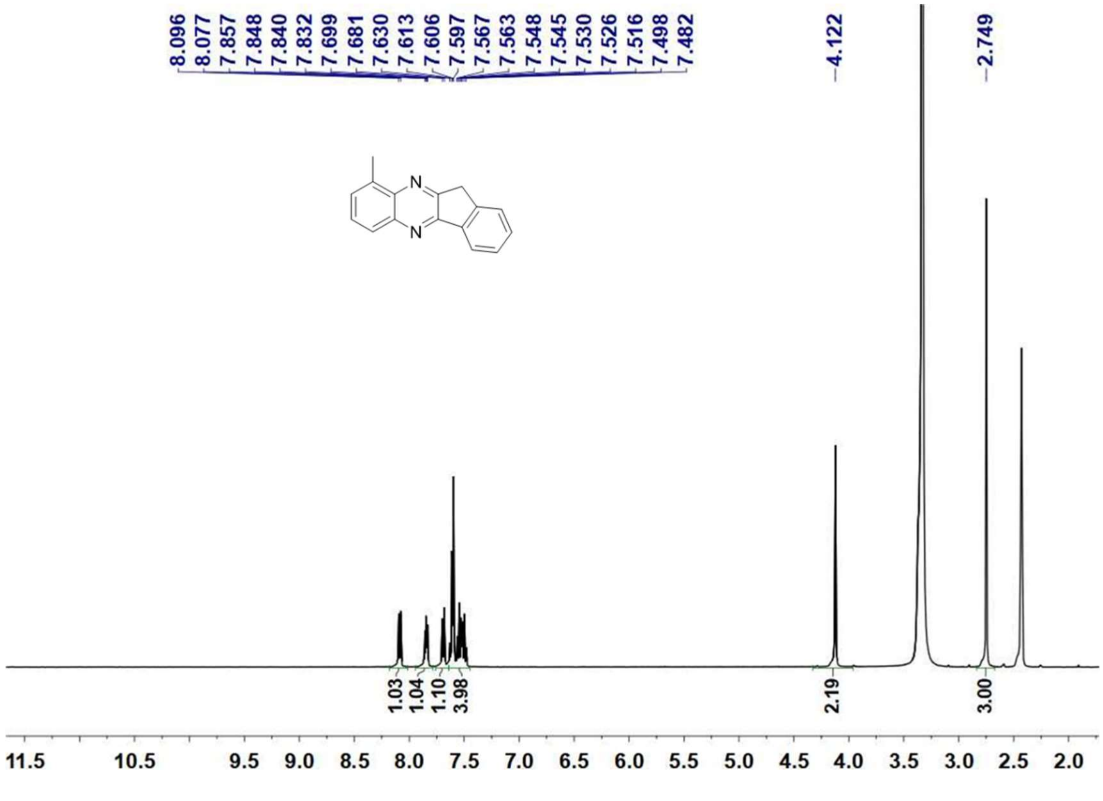

Supplementary Figure 8. ^{1}H NMR spectrum of 9-methyl-11H-indeno[1,2-b]quinoxaline.

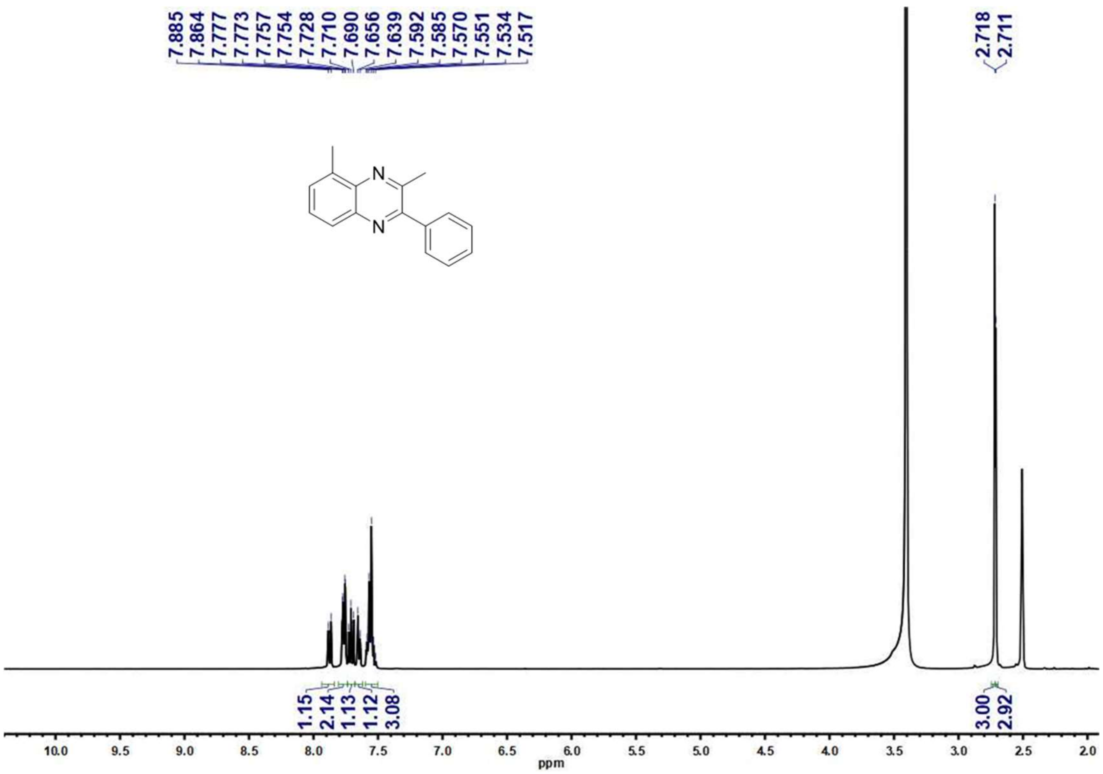

Supplementary Figure 9. ^{1}H NMR spectrum of 3, 5-dimethyl-2-phenylquinoxaline.

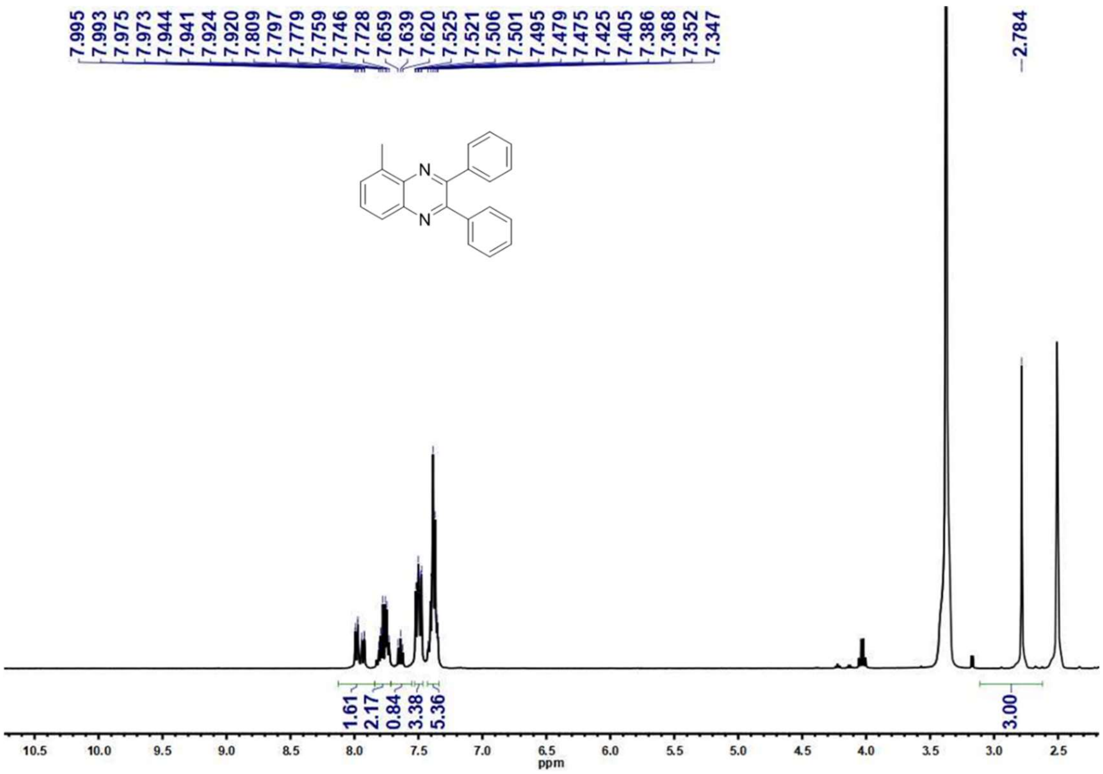

Supplementary Figure 10. ^{1}H NMR spectrum of 5-methyl-2, 3-diphenylquinoxaline.

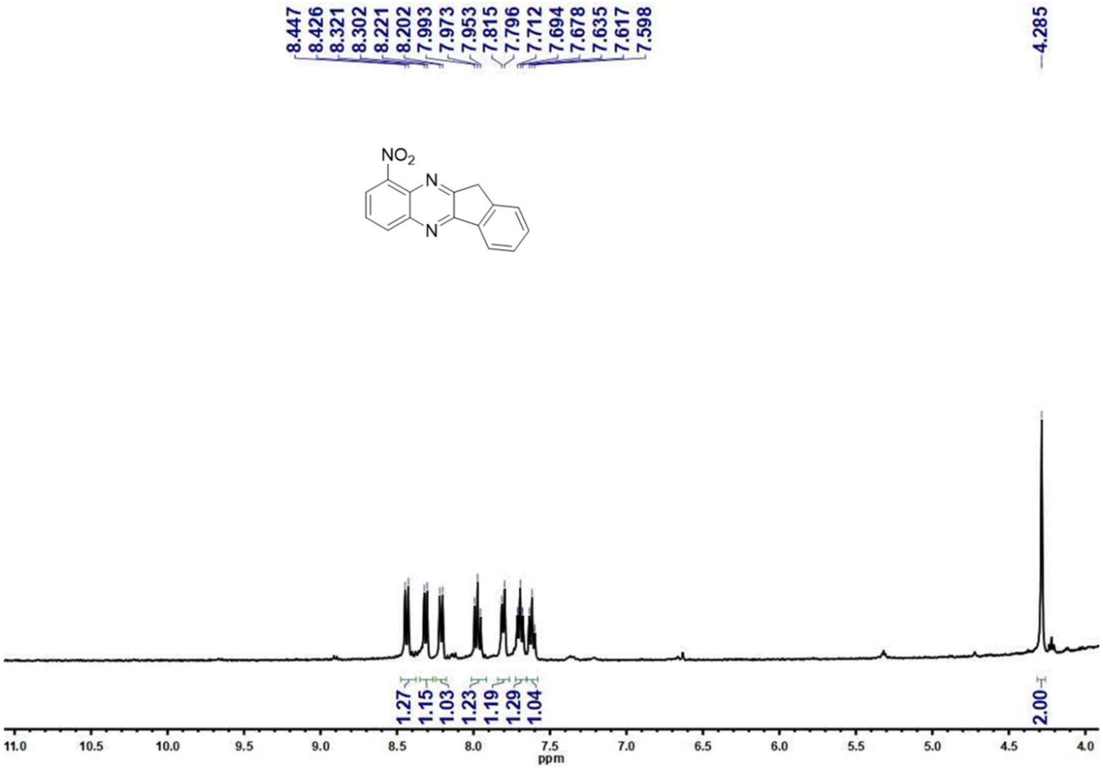

Supplementary Figure 11. ^{1}H NMR spectrum of 9-nitro-11H-indeno[1,2-b]quinoxaline.

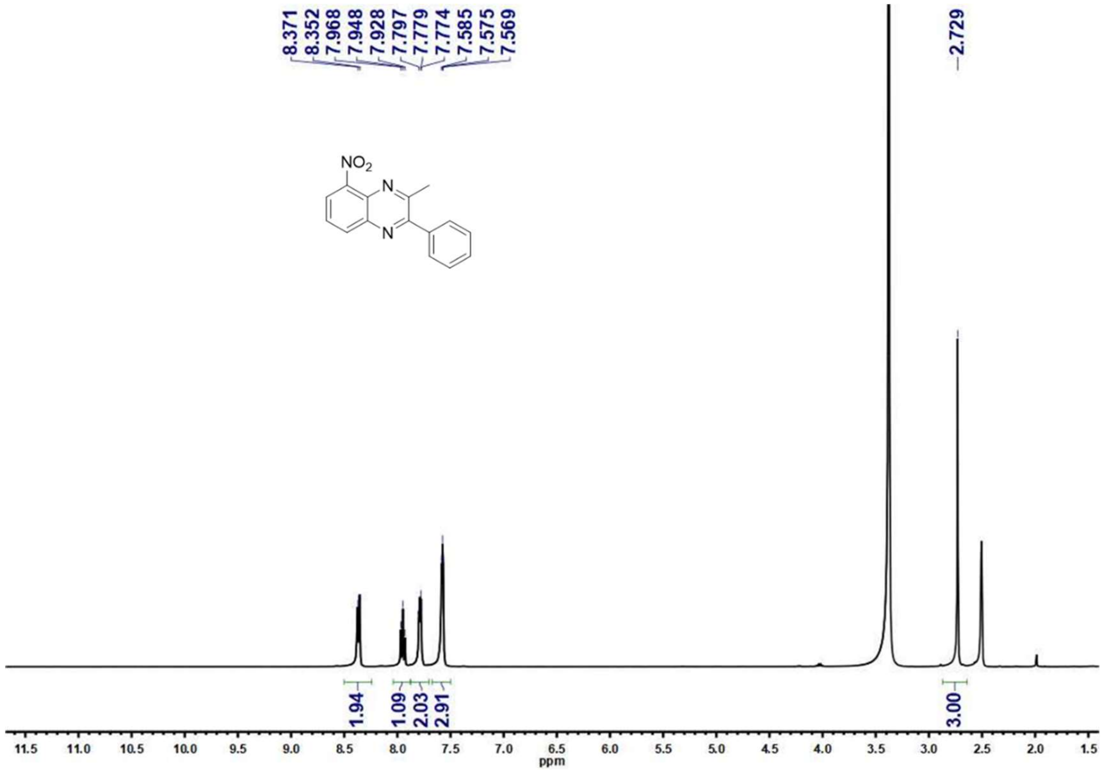

Supplementary Figure 12. ^{1}H NMR spectrum of 3-methyl-5-nitro-2-phenylquinoxaline.

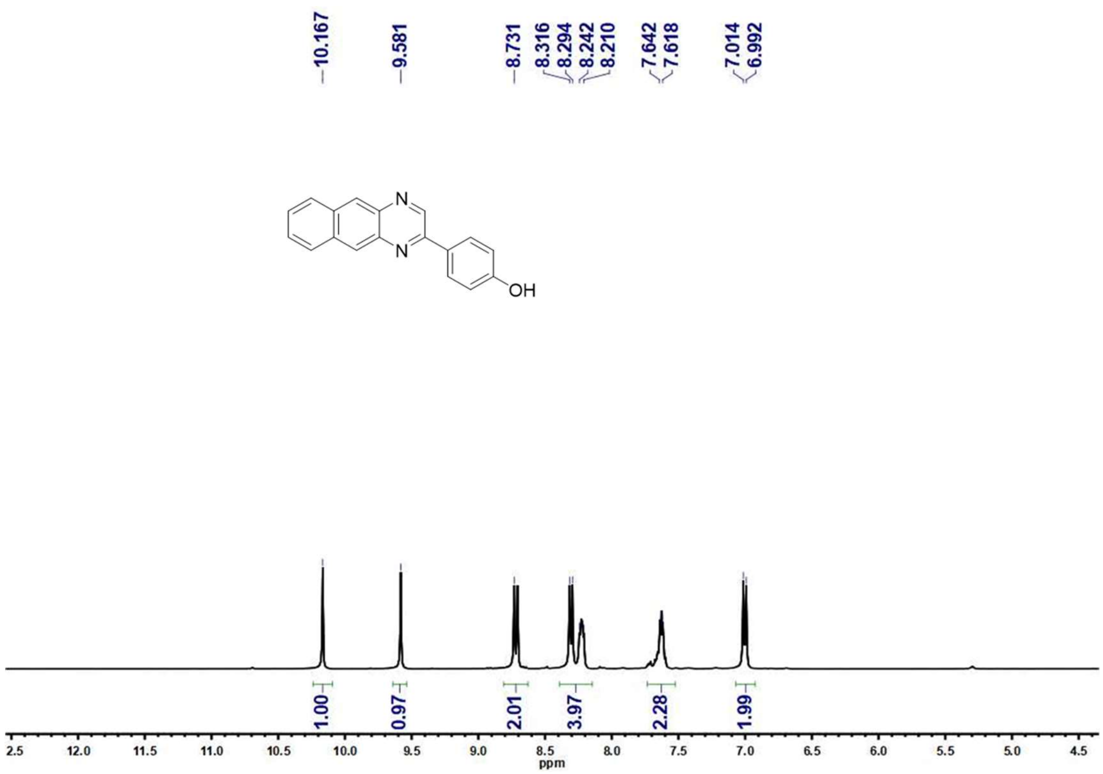

Supplementary Figure 13. ^{1}H NMR spectrum of 4-(benzo[g]quinoxalin-2-yl) phenol.
